# Successful management of an idiopathic first bite syndrome: A case report and review

**DOI:** 10.1002/ccr3.8880

**Published:** 2024-05-08

**Authors:** Behrouz Barati, Matin Ghazizadeh, Arvin Shahzamani

**Affiliations:** ^1^ Department of Otorhinolaryngology, Head and Neck Surgery, Taleghani Hospital Shahid Beheshti University of Medical Sciences Tehran Iran

**Keywords:** case report, idiopathic first bite syndrome, parotid gland, submandibular gland

## Abstract

FBS is associated with surgical interventions or malignancies and could occur idiopathically. Also, this case highlights the successful management of FBS symptoms with pharmacological intervention with gabapentin and carbamazepine.

## 1 INTRODUCTION

A wide range of pathologic conditions can impact the parotid glands, with some being clinically diagnosable and others requiring imaging for diagnosis. First bite syndrome (FBS) is the one that cannot be identified through imaging methods, and its diagnosis solely depends on clinical findings. Netterville initially described FBS in 1998. FBS refers to intense parotid pain experienced upon the first bite of a meal, without any preceding symptoms.^1^, ^2^ The pain gradually diminishes with subsequent bites; thus is most severe during the first bite and the first meal of the day. The prevailing theory regarding the syndrome's pathogenesis, proposed by Netterville et al., suggests that FBS is caused by the loss of sympathetic innervation in the parotid gland.^1^

FBS is commonly considered an early complication following head and neck cancer surgery, occurring in approximately 9.6% of cases. However, primary FBS, which occurs in the absence of a surgical history, is extremely rare and often associated with tumors.^3^, ^4^ If these tumors are malignant and left undetected or untreated, they may infiltrate local tissues and metastasize, significantly impacting the patient's health.^4^, ^5^ Additionally, there are documented instances in the medical literature where the syndrome occurs without any of the aforementioned causes, referred to as idiopathic FBS (IFBS).^6^, ^7^, ^8^ In this report, we present an unusual case of IFBS in a 65‐year‐old male patient.

## 2 CASE HISTORY/EXAMINATION

On September 2023, a 65‐year‐old male patient presented to our institute's outpatient department with a chief complaint of pain in the lower jaw area and submandibular gland lasting for 3 months, specifically while eating. Moreover, palpation or movement of the head did not exacerbate the pain leaving eating the only exacerbating factor. Upon taking a detailed history, the patient reported experiencing acute right dominant bilateral pain (rated 8/10 on the Numerical Rating Scale) upon the first bite of a meal, with the pain gradually subsiding with subsequent food intake and completely disappearing a few minutes after finishing the meal. The patient described the pain as sharp and stabbing, originating from the lower jaw area and submandibular gland and radiating throughout the face. The duration of the pain episodes lasted less than 2 min, and there were no accompanying symptoms. The patient did not exhibit hoarseness, facial sweating following food intake, rhinorrhea, odynophagia, or dysphagia. The patient noted that the clinical symptoms were not dependent on the time of day or positional changes.

Regarding the patient's medical history, there were no underlying diseases, surgical procedures, or current medication use. The patient had not sought any treatment before visiting our facility, and there was no family history of similar symptoms or known allergies. The patient was stable, oriented, and exhibited normal vital signs.

## 3 DIFFERENTIAL DIAGNOSIS, INVESTIGATIONS, AND TREATMENT

During the head and neck examinations, it was observed that the patient wore dentures and did not have any ulcers or deformities. The pharynx, tonsils, soft palate, and tongue were examined regarding ulceration, mass, or any other abnormalities. Parotid glands, nose, temporomandibular joint, and neck showed no abnormalities, and there was no presence of lymphadenopathy. Regional tenderness was absent ruling out glossopharyngeal neuralgia. In the absence of hoarseness, dysphagia, and odynophagia, laryngeal and hypopharynx diseases and Frey syndrome were considered unlikely diagnoses.

**FIGURE 1:**
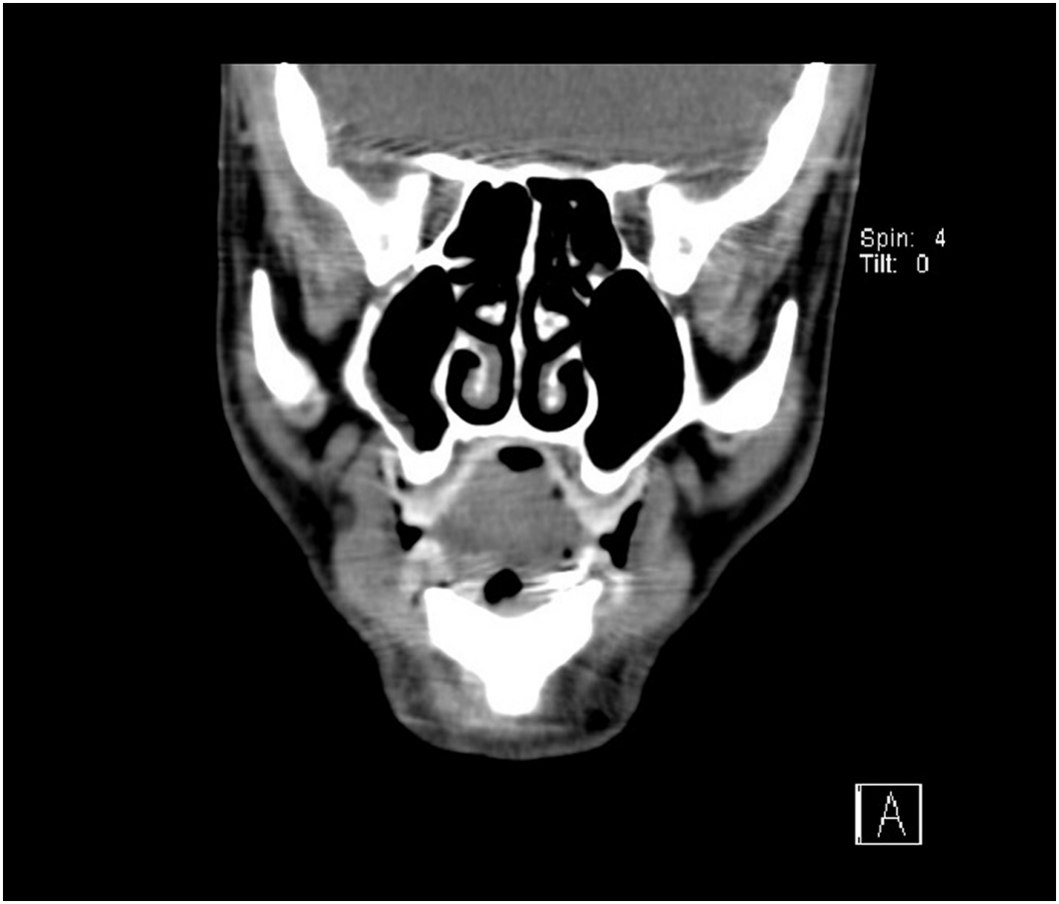
The coronal view of the paranasal sinuses' CT scan was unremarkable, without any space‐occupying lesions.

**FIGURE 2:**
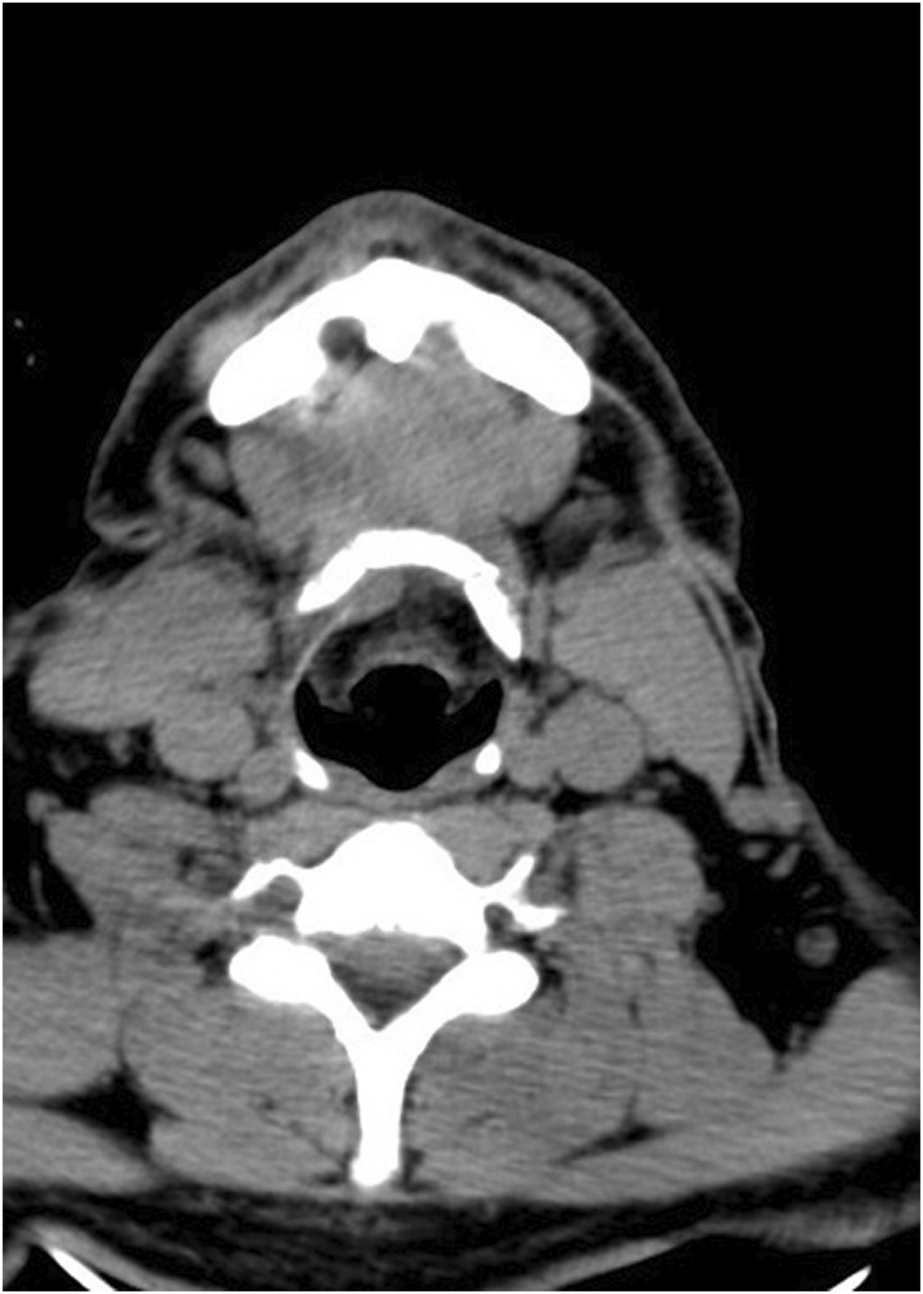
The axial view of neck CT scan shows no space‐occupying lesions or any other pathologic finding.

All laboratory tests conducted on the patient yielded normal results (Table 1). A complete blood count (CBC) was obtained to assess hematologic diseases; viral markers were tested as the affiliated diagnoses might involve salivary glands. Fasting blood sugar was employed to screen for diabetes considering its potential association with FBS.^9^ Screening for thyroid diseases was conducted through thyroid stimulating hormone (TSH). We also ruled out collagen vascular diseases through testing inflammatory and rheumatologic markers. Electrolytes and renal function tests ruled out electrolyte imbalance which could manifest with musculoskeletal pain. Additionally, the CT scan, performed from the skull base to the neck with and without contrast, did not reveal any findings suggestive of head and neck space occupying lesion, mass, sinonasal, or any other pathologic findings (Figures 1 and 2). Based on the patient's history, symptoms, and imaging records, ultimately, the diagnosis of first bite syndrome (FBS) was established.

**TABLE 1:** Laboratory test findings.

Tests	Quantity	Range
WBC	8800	Normal
PLT	223,000	Normal
Hb	14.5	Normal
CRP	2	Normal
ESR	9	Normal
HBs Ag	Negative	Normal
HIV Ab	Negative	Normal
HCV Ab	Negative	Normal
SGOT	25	Normal
SGPT	35	Normal
FBS	94	Normal
BUN	8	Normal
CR	1	Normal
Na	143	Normal
K	4	Normal
Mg	2.1	Normal
Ca	9.2	Normal
RF	Negative	Normal
TSH	1.7	Normal
ANA	Negative	Normal
Anti‐ds DNA	Negative	Normal
C‐ANCA	Negative	Normal
P‐ANCA	Negative	Normal
Anti‐La Ab	Negative	Normal
Anti‐Ro Ab	Negative	Negative

Starting in September, the patient was prescribed gabapentin (100 mg, once daily) and carbamazepine (100 mg, once daily) as medications for neuropathic pain.

### 3.1 Outcome and follow‐up

The patient was scheduled for weekly follow‐up appointments. After 2 weeks, the patient reported a 70% reduction in pain, with a rating of 3/10 on the pain scale. The pain experienced by the patient was further reduced to 2/10 on the pain scale at the 3‐month follow‐up visit. The patient is currently still under follow‐up care.

## 4 DISCUSSION

### 4.1 Review of literature

We identified seven published records related to intermittent first bite syndrome (IFBS). These reports consisted of one case series and five case reports (Table 2). Among these publications, two case reports were originally written in Japanese but had English abstracts available, leading to their inclusion in our review.

**TABLE 2:** Previously reported IFBS cases.

Year of publication	First author	Title	Findings	Treatment/follow‐up
2010	Kurokawa H et al.	IFBS treated with repeated SGB^8^	A case of man in his 30s with a 6‐month history of pain in the parotid region that occurred after the first bite of every meal	The pain completely disappeared after 16 repeats of SGB and did not recur for 2.5 years
2012	Chiba M et al.	A case of IFBS with bilateral onset^10^	A case of 33‐year‐old man, with no prior surgical history, presented with bilateral orofacial pain consistent with FBS	Conservative therapy, including drug therapy and self‐care. At 6 months, his symptoms had almost resolved
2016	Eric T et al.	Letter to the editor: IFBS^6^	A case of 56‐year‐old African American man with severe, bilateral facial pain of 40‐year duration	Oxcarbazepine, 150 mg twice daily, and gabapentin. Currently receiving botulinum toxin A injections to the parotid glands with self‐reported improvements
2018	Masatoshi Chiba et al.	Clinical features of IPP triggered by the first bite in Japanese patients with Type 2 diabetes: A case study of nine patients^5^	Case series of 14 patients with idiopathic parotid pain. Out of which, nine patients were included in this study since they had a history of diabetes, whereas five were excluded as they did not have diabetes or any other underlying pathology	Dietary modifications, to which one patient responded, and pharmacological management consisting of pregabalin or neurotropin
2019	Wemyss C et al.	A case of IFBS responding to carbamazepine^2^	A case of 51‐year‐old male patient with severe, sharp, and unilateral pain occurring only on the first bite of eating	A 200 mg carbamazepine, three times daily 4 months later, complete resolution of symptoms was noted
2019	Hayashi K et al.	IFBS treated with Rikkosan: A case report^7^	A case of 81‐year‐old woman who developed sharp pain in the parotid region with the first bite of every meal	Rikkosan gargle and dietary modifications After 1 year, complete resolution of symptoms with no recurrence was noted
2020	Ramakant S et al.	IFBS^9^	A 35‐year‐old male patient with chief complaint of sharp pain associated bilaterally with parotid on every first bite of the meal	Pregeb M 75 prescribed for 5 months showed an 80% reduction in pain

Abbreviations: IFBS, Idiopathic first bite syndrome; SGB, Stellate ganglion block.

First bite syndrome (FBS) manifests as intense pain in the parotid region, specifically triggered by the initial bite of a meal. Certain gustatory stimuli, such as acids, sourness, spiciness, and sweetness, along with salivation and even the mere thought of food, are believed to exacerbate the condition.^1^, ^2^ FBS is diagnosed by evaluating clinical symptoms and conducting physical examinations. It is essential to differentiate FBS from other facial pain conditions by relying on certain clinical indicators.^11^ The pain experienced in FBS displays distinct features, including sudden and one‐sided onset, reaching its peak during the initial bite of a meal. The pain is specifically located in the preauricular and jaw region and is triggered by salivation. It lasts for seconds to minutes and is characterized by intense, cramping, spasmodic, stabbing, or electric shock‐like sensations. Moreover, a crucial diagnostic criterion for FBS is that no other condition can adequately explain the observed symptoms.^11^, ^12^ Distinctive clinical features of FBS characteristic and pattern of pain and its relation to eating aids to differentiate it from similar entities including laryngopharyngeal reflux and burning mouth syndrome.^13^

The process involves the secretion of parasympathetic neurotransmitters during oral intake, which leads to a cross‐stimulation of sympathetic receptors, resulting in an autonomic imbalance. Consequently, the myoepithelial cells undergo supramaximal contraction due to an aberrant response to parasympathetic stimulation, ultimately causing excessive contraction and subsequent pain in the parotid region upon the first bite.^2^, ^4^ Altogether, the occurrence of pain is attributed to the impairment of sympathetic innervation in the parotid gland. It is hypothesized that excessive activation of the parasympathetic system leads to heightened contraction of myoepithelial cells, resulting in pain.^12^, ^14^ IFBS is commonly associated with a history of previous upper neck surgery, parotid salivary gland tumor, or parotid pleomorphic adenoma involving the parapharyngeal space (PPS), or damage to, or removal of, the cervical sympathetic trunk.^6^, ^7^, ^8^ In this regard, the results of a study showed that the incidence of FBS after parotidectomy was 2% (eight out of 419) during a follow‐up time of 16.5 months. Six out of these eight underwent partial parotidectomy by dissection of the deep lobe of the parotid.^15^ In another study, a rare case of IFBS presented with severe pain and swelling in the parotid region during initial food bites in the absence of prior surgeries or tumors. An extensive literature review found limited cases of idiopathic parotid pain (IPP). This case provides valuable evidence for IPP diagnosis, the first of its kind reported in India. IPP and IFBS terms are used interchangeably.^9^ Beside, another study reported a case that presented bilateral temporomandibular joint (TMJ) pain, self‐resolving closed lock, and limited mouth opening after bilateral replacement, which ultimately was diagnosed with FBS.^16^ In addition, it should be noted that uncontrolled blood sugar levels and diabetic neuropathy could be potential risk factors for IFBS. A case of poorly controlled type I diabetes was reported to experience severe pain in the right condylar process of mandible during the first bite of each meal without a history of head or neck surgery or trauma. This patient was treated with pregabalin and improvement of glycemic control.^17^ On the other hand, FBS could be the initial symptom of another condition. For instance, a case has been reported that presented with preauricular pain after the first bite of a meal, which on further evaluation revealed the diagnosis of squamous cell carcinoma invading the trachea and esophagus.^18^

While symptoms may naturally alleviate over time, some patients require therapeutic intervention due to the severity of their pain. However, currently, there is no established effective treatment for FBS. Diet modifications have not shown proven efficacy.^19^ Nonsteroidal anti‐inflammatory drugs have limited therapeutic benefits.^12^, ^14^ Nonetheless, antiepileptic drugs used for neuralgia and neuropathic pain have demonstrated partial or complete resolution of FBS symptoms (i.e., pregabalin, gabapentin, and carbamazepine).^11^, ^20^ For example, gabapentin therapy gradually and limitedly alleviated the symptoms in a case with FBS following TMJ replacement during the 3‐month follow‐up. However, the patient reported a great improvement in quality of life with gabapentin therapy.^16^ It has been reported that all eight patients with FBS after parotidectomy experienced a resolution of their symptoms without treatment, except for one patient who received gabapentin.^15^ Acupuncture has also shown promising results in two FBS patients by providing complete pain relief.^21^ Although radiotherapy of the parotid gland has been observed to eliminate pain, its use as a therapeutic method for FBS is contraindicated due to its adverse effects. Surgical attempts involving incisions on the tympanic or auriculotemporal nerve to reduce parasympathetic innervation yield unsatisfactory results and pose a high risk of complications.^19^ As another therapeutic alternative, several studies investigated the effects of botulinum toxin for the treatment of FBS. In this line, it has been reported that botulinum toxin type A induces paralysis of the parasympathetic nerves in the parotid gland. This inhibits the release of acetylcholine, a neurotransmitter responsible for triggering intense responses in myoepithelial cells during salivation and mastication. The reduction in acetylcholine release brought about by the toxin leads to decreased contraction of the myoepithelial cells and pathologic secretion from the glands, ultimately minimizing salivation and alleviating symptoms associated with the first bite syndrome.^11^, ^14^ A recent systematic review of eight studies, including 22 patients, was conducted to reveal the effects of botulinum toxin A for the treatment of FBS.^22^ Among the seven studies that reported individual patient outcomes, 16 patients experienced clinical improvement lasting anywhere between 1 and 30 months following the injection.^22^ Notably, all eight patients who received at least 40 U of botulinum toxin A exhibited symptom improvement. Subsequently, 45.5% of patients required a second botulinum toxin A injection due to pain recurrence, with an average time of 3.8 months after the initial injection. Moreover, 38.1% of patients achieved complete resolution of symptoms, with an average duration of 12.1 months.^22^ Importantly, no complications related to the injection procedure were reported in any of the studies, including facial paralysis, infection, injection site reactions, or allergic reactions.^22^ This study represented a 65‐year‐old male patient with lower jaw and submandibular gland pain during meals. The pain resolved after eating. No underlying diseases, surgeries, or medication use were reported. The final diagnosis was IFBS. Gabapentin and carbamazepine were prescribed for neuropathic pain. The pain was reduced by 70% after 2 weeks.

## 5 LIMITATIONS

The findings may have limited generalizability and the conclusions drawn cannot be applied to all patients with FBS. Objective pain scales were not utilized to numerically quantify the degree of pain reduction experienced by the patient, relying only on his subjective reporting on the numerical rating scale. Long‐term follow‐up beyond the initial few weeks posttreatment was not reported, so the sustained effectiveness and durability of the symptomatic relief with gabapentin and carbamazepine remains uncertain. More robust and larger studies are needed to address these limitations.

## AUTHOR CONTRIBUTIONS

**Behrouz Barati:** Conceptualization; investigation; resources; supervision; writing – original draft. **Matin Ghazizadeh:** Conceptualization; investigation; methodology; project administration; supervision; writing – original draft; writing – review and editing. **Arvin Shahzamani:** Methodology; project administration; validation; visualization; writing – original draft; writing – review and editing.

## FUNDING INFORMATION

There is no funding for this article.

## CONFLICT OF INTEREST STATEMENT

The authors state that they have no conflict of interest.

## ETHICS STATEMENT

This article is under The Code of Ethics of the World Medical Association (Declaration of Helsinki) for experiments involving humans.

## CONSENT

Written informed consent was obtained from the patient to publish this report in accordance with the journal's patient consent policy.

## Data Availability

The data that support the findings of this study are available from the corresponding author upon reasonable request.
